# Low Post-Treatment Quality of Life and the High Incidence of Pain Are Common and Significantly Exacerbated in Depressed Head and Neck Patients Treated with Definitive Accelerated Radiotherapy

**DOI:** 10.3390/cancers16010079

**Published:** 2023-12-22

**Authors:** Alicja Heyda, Dorota Księżniak-Baran, Andrzej Wygoda, Krzysztof Składowski

**Affiliations:** 1st Radiation and Clinical Oncology Department, Maria Sklodowska-Curie National Research Institute of Oncology Gliwice Branch, 44-102 Gliwice, Poland

**Keywords:** definitive accelerated radiotherapy, depression, chronic pain, head and neck cancer, quality of life

## Abstract

**Simple Summary:**

Patients treated with definitive accelerated radiotherapy (DART) struggle with low quality of life, pain and persistent treatment-related symptoms even many years after the treatment. Everyday pain in the head, neck and shoulder areas is present among almost all HNC survivors treated with DART. One-third of the examined DART patients were depressed. The depressed group scored significantly worse in most of the quality-of-life subscales and suffered more intense pain than the non-depressed. Head and neck cancer survivors, especially those who are depressed, may require additional psychosocial, physiotherapeutic and medical intervention programs.

**Abstract:**

(1) Background: The goal of this study is to evaluate psychological tolerance and health-related quality of life (QOL) in head and neck (HN) cancer patients treated with definitive accelerated radiotherapy (DART). (2) Methods: 76 recurrence-free patients eligible for the study, who were treated with DART in the CAIR-2 phase III clinical study (median of follow-up = 47 months), completed EORTC QLQ-C30 with the H&N35 module, Hospital Anxiety and Depression Scale (HADS) and Visual–Analog Scales (VAS) of pain in HN and the neck/arm areas. (3) Results: The most dominant symptoms measured with QLQ-C30 were as follows: fatigue (44/100), sleeplessness (39/100), financial problems (38/100) and pain (32/100). Within the H&N35, the highest scores were reported on the subscales of sticky saliva (60/100), mouth dryness (65/100) and increased intake of painkillers (50/100). Pain (VAS) was reported by 87% (HN area) and 78% (shoulder area) of the patients, with a mean score of 3/10. One-third of the patients reported depressive moods (HADS ≥ 15 points) with an average score of 12.5/42 p. The depressed group, who smoked more as compared to the non-depressed group before DART (96% vs. 78%) and required steroids treatment (85% vs. 58%) during DART, also scored significantly worse on 23 of the 35 subscales of QLQ-C30 and H&N35 and experienced more intense pain (VAS). Women and less-advanced patients scored better in several aspects of quality of life. (4) Conclusions: Patients treated with DART struggle with low quality of life and persistent treatment-related symptoms including constant pain. HNC survivors, especially those who are depressed, may require additional psychosocial, rehabilitation and medical intervention programmes.

## 1. Introduction

Most quality-of-life studies of head and neck cancer survivors showed that patients struggle with distress, depression, pain and a large variety of persistent cancer-related symptoms. Quality of life domains like the global quality of life, pain, eating and speech problems [1,2,3,4] were associated with HN survival. A shorter overall survival rate is also present in the cases of pre-existing depression or depressive states [5,6].

The 3–40% prevalence of depression after treatment of HNC has been noted in various studies [7]. Depression and distress were found during the first year after treatment [8] and during the long-term follow-up period [9,10,11]. The disease’s state of advancement, being unmarried, and helpless/hopeless coping strategies were predictors of depressed moods in head and neck cancer survivors [12]. More depressive symptoms in HNC survivors were also found in groups with moderate to severe dysphagia [13] and in patients with gastrostomy tubes [14].

Persistent or worsening treatment-related symptoms after treatment of HNC were reported in various prospective studies [15,16,17,18,19]. In a large cross-sectional study of 640 HNC cancer survivors (median of follow-up 3–4 years) dry mouth, sticky saliva and teeth problems were the most severe symptoms reported by the patients [20]. Intimacy problems and sexual dysfunctions were also reported by 25% of patients after primary HNC treatment [21].

The modification of the standard radiotherapy (once a day for five days a week, fractionation dose of 1.8 to 2 Gy, for five to seven weeks) in head and neck cancer patients entails an increase in the severity of acute (observed during and shortly after treatment), radiation-induced toxicities, mainly mucositis. However, in most reports, the incidence and intensity of late complications (xerostomia, tissue fibrosis, taste impairment) remain similar in patients after standard radiotherapy and those treated with the use of various methods of modified standard treatment (hyperfractionation, accelerated fractionation, accelerated hyperfractionation) [22,23,24,25,26].

The meta-analysis of 6515 head and neck cancer patients from 15 different studies who were treated with different types of altered fractionated radiotherapy showed that this method can improve both survival rates and locoregional control [25]. Although hyperfractionated or accelerated radiotherapy (AR) with or without total dose reduction gained a lot of scientific attention, only a few studies have explored the effects of altered fractional radiotherapy on detailed quality of life and/or psychological tolerance using various measurement tools. 

Several existing studies on hyperfractionated radiotherapy using different measurement methods showed no differences in most dimensions of post-treatment QOL between conventionally and hyperfractionated groups [11,27,28,29]. A longitudinal study comparing accelerated versus conventional radiotherapy in 750 HNC patients showed that the AR group reported the worst quality of life at the end of radiotherapy; most differences were observed at three months later, while several others were observed at six months and five years later. Oral functions did not reach the baseline level in both groups [30]. Detailed quality of life in definitive accelerated radiotherapy was also described in another two studies [31,32], but they were difficult to analyse due to group heterogeneity (several patients received chemotherapy or/and underwent neck dissection.

It is significant that the late toxicities of radiotherapy can be assessed by using various scales, such as LENT/SOMA, RTOG/EORTC and NCI-CTC. Therefore, the observed severity of late complications may vary depending on the selected reporting method [26]. Subscales in the above-mentioned scales do not consider psychological and socio-economic factors. After all, the quality of life after radiotherapy is a result not only of observed morphological or functional defects but also of accompanying diseases (somatic and mental), lifestyle (nutrition, smoking) or the already-mentioned aspects related to the economic status of patients.

Although there are a few publications on the subject of the detailed quality of life after hyperfractionated radiotherapy of HNC patients, until now no such publications have focused exclusively on definitive accelerated radiotherapy in HNC without any chemotherapy or surgery. There are no QOL studies concerning further psychological evaluation that is more detailed than basic QOL in HNC patients treated with DART. The purpose of this study is to examine the quality of life and psychological tolerance of patients who were treated with definitive accelerated radiotherapy.

Observer-based scoring systems like the DAHANCA toxicity score or the CTCAE do not recognize many symptoms of HN patients, unlike the quality-of-life scales which refer to their own complaints [33]. Therefore, detailed QOL research in head and neck cancer patients requires the usage of patient-based surveys, especially in clinical trials. Quality-of-life research on head and neck cancer patients may currently be measured with 13 measurement tools designed exclusively for HNC patients [34]. Researchers also use general quality-of-life questionnaires such as the EORTC QLQ-C30 for cancer patients or SF-36 for the general population.

## 2. Materials and Methods

This is a cross-sectional survey of HNC patients with follow-ups lasting longer than 12 months. All patients included in this study took part in the CAIR-2 phase III clinical trial [35] guided in the First Radiotherapy Department, Comprehensive Cancer Center Marie Skłodowska-Curie Institute of Oncology Gliwice Branch, Poland. All patients with squamous cell carcinoma of head and neck in Stage T(2–4)N(0–1)M(0) received two definitive radiation treatments: accelerated fractionation 7 days a week including weekends (CAIR) or 7 fractions 5 days a week (Concomitant Boost, CB). The fractionation parameters remained within the range of 66.6–72 Gy of the total dose: 37–40 fractions and 37–40 days. Most of the patients received conformal radiotherapy (2D—14%, 3D—45%, IMRT—25%, 3D + IMRT—16%); see Table 1. The quality-of-life outcomes of the DART group were compared to the results of 640 HNC patients with similar median follow-ups [20] who completed QLQ-C30 and H&N35 and were treated with different types of radiotherapy with doses ranging from 54 to 79 Gy.

The results show that weekend breaks have no impact on treatment outcomes if radiotherapy is accelerated [33]; therefore, using the same dose and treatment time, the quality-of-life scores of the CB and CAIR groups were analysed as one DART group altogether. For patients’ initial characteristics, see Table 1.

Out of 184 patients who were treated with DART between the years 1995–2006, 24 of them were deceased at the time of the study. All the patients who agreed to take part in the study signed written consent forms and agreed to fill out the set of psychological and QOL tests. Seventy-eight patients were sent back or brought filled questionnaires. Two patients were excluded from the study because of secondary cancers. The follow-up time median was 47 months (12 to 158 months) after completing the treatment (ibid.). 

The instruments selected for this study are well-known tools for measuring the quality of life, pain and psychological status of patients. The health-related quality of life of HNC patients was measured using the EORTC QLQ-C30 with the head and neck module H&N35 [36,37]. The scores of QLQ-C30 and H&N35 ranged from 0 to 100. The higher the scores on the functional subscales of QLQ-C30, the better the patient’s functioning. Scores of H&N35 are symptom scales, so a higher result means that the symptom is more severe.

The baseline T and N statuses and the patient’s gender were used as independent data for comparing the intergroup differences in QOL and treatment-related data. A subgroup of 359 patients (67%) received the dose > 70.2 GY; 371 patients were treated by 2DRT and 269 patients by conformal RT (3DCRT: 127 patients, IMRT: 142 patients). The clinically significant differences of 10 points and more [37] between the groups in all QLQ-C30 subscales were analysed.

Two VAS scales, ranging from 0 to 10 points [38] were used to evaluate pain intensity in the head/neck areas and, separately, in the shoulder areas. 

Depression and anxiety were examined using HADS: the Hospital Anxiety and Depression Scale [39,40]. The HAD scale is recognized as a sensitive instrument for the screening of depressive symptomatology in HNC patients [41] The sum of anxiety and depression subscales were also examined; a total of 15 points was the cutoff score for recognizing major depression in HNC patients (ibid.). The patient’s QOL and VAS outcomes and their initial characteristics including age, gender, marital status, education, smoking, TNM, presence of cancer symptoms before diagnosis, time from diagnosis to treatment ZUBROD, dose, irradiation method, corticosteroids and analgesics intake, dysphagia (see Table 1) were analysed using the HADS cutoff score. Data were analysed using the non-parametric Mann-Whitney U test. 

## 3. Results

Patients scored highest in the role (85/100), social (79/100) and cognitive functioning (73/100) subscales. Fatigue (44/100), insomnia (39/100) and financial problems (38/100) were the most severe symptoms of QLQ-C30 reported by the patients. From theH&N35 scores, mouth dryness (65/100), sticky saliva (61/100) and painkiller intake (50/100) were the most severe symptoms: see Table 2. As compared to the large HNC survivor group who completed radiotherapy with similar follow-up times [20], the DART group scored significantly worse with 10 points or more, a clinically significant difference, ref. [42] in physical, emotional and financial functioning; the intensity of fatigue; insomnia; sexuality; dry mouth; sticky saliva and coughing. 

Inter-group differences were analysed in all treatment-related, biological, QOL and psychosocial variables (see Table 1) using such grouping factors as gender, T and N stage and cutoff score for depression (clinically relevant overall HADS score ≥ 15 points). All statistically significant results are presented in Table 3. The results for all groups are presented as means, not medians, due to the characters of the single-item questions of QLQ-C30 and H&N35 modules in which the median is always 1 or 0.

Compared to the male patients, the female patient initially lost less weight during radiotherapy. They also reported lower frequencies of pain in the head and neck areas as measured by the H&N35 subscale (*p* < 0.05), fewer swallowing problems (*p* < 0.01) and fewer problems with eating in the public (*p* < 0.05). Men reported more severe appetite loss (*p* < 0.05) and more problems with sticky saliva (*p* < 0.05, Table 3). 

A group of 42 patients with T2 and a group of 34 patients with T3 and T4 categories were analysed. Patients with more advanced T categories reported significantly worse cognitive functioning (*p* < 0.05), more problems with opening their mouths (*p* < 0.01), more problems with speech (*p* < 0.02) and more difficulty with social eating (*p* < 0.02). They also reported more frequent pain in the head and neck areas (*p* < 0.05). Patients with N0 advancement (n = 52) were compared to the N1 group (n = 24). The nodal advancement was associated with worse cognitive functioning (*p* < 0.05), problems with the opening the mouth (*p* < 0.02) and the feeling senses (*p* < 0.01), trouble with social eating (*p* < 0.01) and dysphagia (*p* < 0.05) during the treatment (see Table 3). 

The intensity of pain in the head and neck (HN) areas scored 3.4 on the VAS scale (1–3 points pain on 0–10 VAS is considered a weak pain). The pain in the shoulder area for both groups scored 2.9 points. Only 10 patients (13%) did not report any pain. This suggests that the majority of patients experienced some kind of HN pain. Very intense HN pain (7–10 p) was reported by 10% of the patients. Pain in the shoulders was reported by 78% of patients. In most cases, the intensity of pain was weak or moderate (see Table 4).

The analysis revealed a low average level of depression and anxiety in the whole group (below 8 points: 6/21 p and 7/21 p, respectively). The clinically significant level of the overall HADS score, 15 points and more [36], signified that 27% of the patients possibly were depressed. The mean result was 12.25 points—below the cut-off score (Table 4).

Comparing the depressed group (27%) to the non-depressed revealed many significant differences within variables like smoking before treatment, corticosteroids intake during and directly post-treatment, quality of life data, emotional states and traits and pain. The patients reported worse social, emotional and cognitive functioning and lower global health status. The intensity and frequency of pain were greater in the depressed group. Treatment-related symptoms, except for pain, were also more frequent in the depressed group and included fatigue, insomnia, appetite loss, swallowing, speech, social eating and contact, difficulty opening the mouth, mouth dryness, sticky saliva, deterioration of sexuality, coughing, painkiller intake and weight loss; the depressed group scored worse in 23 out of 35 subscales in the QLQ-C-30 and H&N35. The depressed patients who smoked before DART treatment were required to take corticosteroids during and right after DART more often than the non-depressed. The patients’ initial biological characteristics and medical statuses 3.5 y after the survey timeline did not differ in either subgroup. Statistically significant differences are listed in Table 5.

## 4. Discussion

The experience of persistent pain dominates among the other symptoms, despite practices including the use of painkillers and attending follow-up visits. A large majority of the DART-treated patients reported pain in the head/neck and shoulder areas (87% and 78% respectively). Other studies on the quality of life in HNC survivors also showed that pain and other treatment-related symptoms persist or worsen during follow-ups that last longer than 1 year [15,17,18,19]. The DART group’s reported pain, both in the HN areas and the arm and shoulder areas after cancer treatment, which persisted or possibly increased over the years post-treatment. A meta-analysis of 82 studies evaluating pain in HNC patients treated according to various schemes estimated that the incidence of pain occurred in 57% of patients before treatment and in 42% after treatment [43]. This result shows a much better outcome than the results of DART patients, in which the intensification of pain in the head and neck areas concerned almost all the examined patients. The intensity of pain in the analysed DART group was also found to be higher than the intensity in another study of patients with HNC’s pain severity both before and after radiation treatment with VAS, as reported up to 24 months after the treatment [44]. 

Persistent treatment–related symptoms are consistent with the already existing research on QOL in H&N patients. Patients scored high on QOL dimensions such as functioning in their life roles as well as in social and cognitive areas. Fatigue, insomnia and financial difficulties were the most common symptoms from the QLQ-C30 subscales that were reported by patients treated with DART. Sticky saliva, mouth dryness and painkiller intake had the highest scores out of all subscales from the H&N35 module. Generally, the patients functioned better in social, emotional and cognitive dimensions as compared to treatment-related ailments. 

As compared to a large sample of HNC survivors from the Leung et al. study (see Table 2) with a similar median of follow-up range but with several different irradiation types [20], the DART patients reported worse functioning in most of the analysed QoL dimensions except for global health status, social functioning and mouth opening. The DART patients scored significantly worse—with more than 10 points of variance [38]—on the subscales of physical functioning, emotional functioning, fatigue, pain, insomnia and financial difficulties. The largest, clinically relevant differences within treatment-specific symptoms (H&N35) between the DART group and the other group were found in the subscales evaluating sticky saliva, dry mouth, coughing and diminished sexuality. 

These results might correspond to a quality-of-life study comparing accelerated fractionation to conventional by Nyqvist et al. [30]. Treatment time reduction that results in dose intensification is directly related to persistently low quality of life outcomes.

The reasons for gender differences in quality of life and psychological outcomes remain unclear. Different QOL studies present contradictory results when comparing the QOL of men and women treated for HNC [45,46]. 

Inter-group differences depending on T and N statuses seem to be mostly associated with more intensive treatments in the cases of more advanced diseases. This study has revealed a relationship between primary nodal and tumour advancement and worse cognitive functioning, which could be explained by fatigue and the intensity of xerostomia related to larger volumes of irradiation. In a prospective study of HNC patients undergoing radical treatment, significant associations were found between the RTOG-measured xerostomia and overall quality of life (effect size = 0.07, *p* < 0.001). Significant relations were observed between global QOL and all functioning scales, including the cognitive functioning scale, fatigue and insomnia. The results demonstrate the important effect of radiation-induced xerostomia on QOL. Although the incidence of grade ≥2 RTOG-xerostomia gradually decreased, its effects on QoL increased over time. This finding highlights the importance of the prevention of xerostomia [47]. In another study, post-treatment (median: 7 years) neuropsychological measurement of the cognitive functions of HNC patients revealed worse cognitive performance in the areas of episodic memory and the speed of information processing. Cognitive function assessed by neuropsychological testing was related to the self-perceived worsening of cognitive functioning, although it was not related with depressive symptoms. Cerebral infarctions on the MRI were correlated with cognitive function contrary to cerebral white matter hyperintensities and brain volume. In general, the subject of cognitive decline after radical HNC treatment is still not well-studied but deserves attention [48].

Although the mean results on depression, anxiety or overall HADS score were below the clinically relevant score, one-third (27%) of the patients scored more than 15 points above the major depression cutoff score. This shows that one-third of this group had poor psychological outcomes. The results comparing the depressed and non-depressed groups revealed many significant differences in quality-of-life subscales, emotional states and pain levels. The deterioration of quality of life and increased pain levels are linked to cancer-related depression [49,50,51].

The more common corticosteroid intake during DART in the depressed group suggests that their treatment caused more intense symptoms of acute mucositis and might have been more difficult to bear. The previous intake of corticosteroids by cancer patients is linked to depressive symptomatology following such treatment [52]. Patients with moderate- and high-stage head and neck cancer undergoing radical radiotherapy develop a clinically significant acute post-radiation reaction of the mucosa (mucositis). During treatment, acute mucositis is particularly severe in patients undergoing accelerated radiotherapy. Such patients receive irradiation to a larger volume of tissue, which consequently leads to the acute reaction affecting larger mucosa surfaces. The symptomatic treatment of acute mucositis was implemented as a gradual pharmacotherapeutic intervention. Its treatment begins with drugs that act locally and systemically and are prescribed in the following order: local analgesics, systemic analgesics and non-steroidal anti-inflammatory drugs, steroidal anti-inflammatory drugs and opioids. The use of steroid drugs significantly reduced the severity of acute radiation reactions and, in many cases, allowed for the completion of radiotherapy as planned, without radiotherapy treatment breaks [35,53]. Despite the side effects of steroid therapy, the benefits resulting from their use outweighed their side effects. The steroid treatment was usually short-term and usually did not exceed three weeks (see Table 1).

It is significant that the depressed DART group admitted to smoking more often before the treatment. The prevalence of depressive symptoms is also often related to the fear of disease recurrence in head and neck cancer survivors [54,55]. The level of cigarette consumption was a predictor of psychological distress 15 months after treatment in head and neck cancer survivors. Smoking was also linked to baseline distress and fear of recurrence [56].

A review of the existing research on the comorbidity of pain and depression showed that pain and depression occur simultaneously in approximately 35% of patients (22–49%) [57]. A longitudinal study examining the relationship between the occurrence of pain and mood disorders in cancer patients also showed the interdependence of both symptoms. Patients who responded well to analgesic treatment (61%) had significantly lower HADS scores within a few weeks of treatment, unlike the patients who had no improvement in pain [58].

## 5. Conclusions

All results from this cross-sectional study allow limited conclusions to be drawn due to its cross-sectional nature, which prevents the assessment of changes over time. The group itself cannot be representative of all patients treated this way, as the sample is relatively small. Nevertheless, patients treated with DART struggle with low quality of life and persisting treatment-related symptoms including constant pain. Pain and multifaceted psychological and somatic ailments dominate the profile of self-rated quality of life.

The intensity of distress/depression strongly affects patients’ perception and reception of post-treatment symptoms as well as their general functioning. Both facts are consistent with the quality-of-life research conducted on HNC survivors after different kinds of treatment. The presence of depression in DART-treated patients co-exists with intensive post-treatment oral symptoms, weight loss and lower QOL. The level of self-rated depression and distress might be the factor that distinguishes between the patients who can receive standard care and those who need non-standard, more intensive care. Screening for distress/depression should become part of conventional follow-up visits after DART. HNC survivors who are depressed may require additional psychosocial, physiotherapeutic and medical intervention programmes.

Recent recommendations by the Association for Clinical Oncology and the Society of Integrative Oncology (ASCO and SIO) for post-treatment depression in cancer patients include mindfulness-based interventions as integrative interventions that have a high level of evidence. This same recommendation refers to treating anxiety in cancer survivors [59]. A systematic review based on 21 studies from 1980 to 2017 of depression-related psychological interventions in head and neck cancers has revealed the greatest empirical support for psychotherapy (CBT) and psychoeducation [60].

## Figures and Tables

**Table 1:** Initial patients and treatment characteristics.

Patients & Treatment Characteristics	
CAIR-2 phase III clinical trial participants	184 pts
Deceased at the time of the study	24
Eligible for the study	160
Patients who filled QOL tests	78 (2 excluded due to secondary cancer)
Tests included into study	76
Response rate	48%
Age	Median: 64 y
Gender	men—70%,
	women—30%
Marital status	married—79%,
	widowed—13%,
	single—6.7%,
	divorced—1.3%
Education and overall years of education)	primary school (8 y)—20%,
	secondary school (11 y)—39%,
	high school (12 y)—33%,
	master’s degree (16 y)—8%
Smoking tobacco before DART	84%
Cancer symptoms before diagnosis	median: 5.5 months (1–84)
Time from diagnosis to treatment	median: 49 days (15–188)
Tracheostomy before DART	0
Feeding tube during DART	4%
Neck dissection or surgery before DART	0
ZUBROD status before DART	0—80%, 1–18.7%, 2–1.3 %,
ZUBROD status after DART	0–70%, 1–28.7%, 2 –1.3%
Weight loss during the treatment	Mean = −2.4 kg (−12.3 kg +7.9 kg)
Analgesics type during and after DART	no analgesics—3%,
	local—9%,
	NSAIDs + tramadol—72%,
	opiates—16%
Analgesics intake (days)	median: 21 days (5–173)
Dysphagia during and after DART	no dysphagia—41%,
	mixed food—51%,
	liquids only—8%
Dysphagia (days	median: 20 days (6–189)
Corticosteroids intake during and directly after DART	68%
Corticosteroids intake (days)	median: 20 days (1–50)
Length of follow-up	Median: 47 months (12–158)
TNM classification	
T	T2—53%, T3—23%, T4—24%,
N	N0—69%, N1—31%
Location:	hypopharynx—8%,
	oropharynx—25%,
	larynx—53%
	oral cavity—14%
Technique	2D—14%, 3D—45%, IMRT—25%, 3D + IMRT—16%
Fraction schedule	1.8 Gy/fx
Total dose	66.6–72 Gy
No of fractions/days	37–40 fractions and 37–40 days

**Table 2:** Results of the QLQ-C30 and H&N35 of DART survivors with a follow-up median of 47 months, compared with 640 HNC survivors with a similar median of follow-up who received various radiotherapy treatments from Leung et al. [20]. Arrow symbols—DART group scored 10 points or more worse ↓ or better ↑ than the compared group.

QLQ-C30 and H&N 35	DART Survivors		Leung et al., 2011		Clinically Significant Difference—10 Points or More between the Means
Subscales (Range 0–100)	n = 76 (Sexuality Scale n = 63)		n = 640		
	Median of Follow-Up: 47 m.		Median of Follow-Up: 51 m		
	Mean (sd)	Median	Mean (sd)	Median	
**QLQ-C30 Functional scales:**					
Global health status					
Physical functioning	58.6 (23.1)	58.3	54.6 (19.9)	50	↑4
Role functioning	68.9 (20.2)	68.7	84.5 (16.5)	86.7	↓15.6
Emotional functioning	85.7 (19.4)	100	86.4 (21)	100	↓0.7
Cognitive functioning	62.9 (23.2)	66.7	77.9 (20)	75	↓15
Social functioning	73.1 (21.2)	66.7	78.4 (19.8)	83.3	↓5.3
**QLQ-C30 Symptom scales:**	80.1 (25)	83.3	75.1 (24.2)	66.7	↑5
Fatigue FA					
Nausea and vomiting NV	43.6 (25.6) 8.9 (15.1)	44.3	29.2 (20.04)	33.3	↓14.4
Pain PA	32.40(28.4)	0	9.3 (17.5)	0	↑0.4
Dyspnea DY	20.4 (26.2)	33.3	21.9 (22.4)	16.7	↓10.5
Insomnia	38.8 (33.8)	0	15.2 (21.16)	0	↓5.2
Appetite loss AP	29.7 (26.9)	44.4	26.3 (25.9)	33.3	↓12.4
Constipation CO	22.5 (28.2)	30	19.9 (24.6)	0	↓2.6
Diarrhea DI	10.3 (15.3)	0	19.1 (23.7)	33.3	↓3.4
Financial difficulties FI	37.6 (38.8)	0	14 (19.9)	0	↓3.7
		33.3	26.4 (27.1)	33.3	↓11.2
**H&N35 (symptoms scales)**					
HN pain HNPA	28.2 (21.5)	25	19.1 (20.6)	16.7	↓9.1
Swallowing problems HNSW	26.7 (25.4)	16.7	30.2 (24.4)	25	↓3.5
Senses problems HNSE	31.2 (30.6)	33	26.9 (27.7)	25	↓4.39
Speech HNSP	28.9 (23.7)	22	28 (25.9)	22.2	↓0.9
Social eating HNSO	28.7 (26.6)	25	28.5 (26.9)	25	↓0.9
Social contact HNSC	19.6 (19.9)	13	20.4 (23.5)	13.3	↓0.8
Less sexuality HNSX	38.6 (34.9)	33	26 (27.7)	33.3	↓12.6
Teeth HNTE	26 (36.5)	0	38.9 (29.8)	33.3	↑12.9
Opening mouth HNOM	27.4 (37.8)	0	32.8 (31.6)	33.3	↑5.4
Dry mouth HNDR	64.8 (32.3)	66.7	48.3 (31)	33.3	↓16.5
Sticky saliva HNSS	60.7 (34.8)	66.7	40.7 (30.6)	33.3	↓20
Coughing HNCO	42.7 (31)	33.3	29.3 (25.4)		↓13.4
Feeling ill HNFI	36.5 (32.5)	33.3	29.3 (25.3)		↓7.2
**H&N35 single items**					
			Not analysed	-	
Painkillers HNPK			-	-	
Nutritional supplements HNNU	50 (50)	50	-	-	-
Feeding tube HNFE	18.9 (39.4)	0	-	-	-
Weight loss HNWL		0	-	-	-
Weight gain HNWG	0	0	-		-
	24.3 (43)	0	-		-
	36 (48.3)				-

**Table 3:** Statistically significant differences in mood, pain, QoL and medical data depending on the gender and the T and N status.

Variables	Test Type (if Relevant)	Grouping VARIABLE vs. Mean/Median Results for Analyzed Groups			*p* Value Mann-Whitney U-Test
		**Gender**			
		**men (M/ME)**	**women (M/ME)**		
Appetite loss AP	QLQ-C30	33/33.3	23/0		<0.05
Pain of H&N area—HNPA	H&N35	32/33.3	20/16.7		<0.05
Sticky saliva HNSS	H&N35	32/33.3	16/8.3		<0.05
Swallowing problems HNSW	H&N35	67/66.7	47/33.3		<0.01
Social eating HNSO	H&N35	33/25	20/16.7		<0.05
Weight loss during RT (kg)	-	−2.9/−2.7	−1.2/−0.8		<0.05
		**T-stage**			
		**T2 (M/ME)**	**T3 + T4 (M/ME)**		
Cognitive functioning CF	QLQ-C30 H&N35	78/83	66/66.7		<0.01
					<0.01
Opening mouth HNOM	H&N35	17/0	42/33.3		
Pain of H&N area—HNPA	H&N35	23/25	35/33.3		<0.05
Speech problems HNSP	H&N35	25/22.2		34/33.3	<0.05
Social eating HNSO		23/16.7		37/33.3	
		**N-stage**			
		**N0 (M/ME)**		**N1 (M/ME)**	
Cognitive functioning CF	QLQ-C30	77/83.3		64/66.7	<0.05
Opening mouth HNOM	H&N35	18/16.7		45/33.3	<0.05
Senses problems HNSE	H&N35	24/16.7		44/37.5	<0.01
Social eating HNSO	H&N35	23/16.7		40/33.3	<0.01
Dysphagia during DART (mixed food or liquids only)	-	58%	87%		<0.05

**Table 4:** The mood (HADS) and intensity of pain (VAS) in the DART group.

Mood (HADS)	Results	Std. Dev.	Median/Quartiles
Depression (0–21 p.)	mean = 6	4.53	5 (2–9)
Anxiety (0–21 p.)	mean = 7	4.41	6 (2.5–10)
Sum of depression + anxiety	mean = 12.5	8.37	10.5 (6–18)
% of possible major depression outcomes (≥15 points)	27%		
**Intensity of Pain (VAS, 0–10 p.)**			
Pain of the head and neck area	mean = 3.4	2.46	3 (1–5)
no pain (0 points)	13%		
weak pain (1–3 p)	37%		
moderate pain (4–6 p)	40%		
strong pain (7–10 p)	10%		
Pain of the shoulder area	mean = 2.9	2.44	3 (1–5)
no pain (0 points)	22%		
weak pain (1–3 p)	41%		
moderate pain (4–6 p)	32%		
strong pain (7–10 p)	4.5%		

**Table 5:** Significant differences between the depressed and non-depressed groups (Mann-Whitney’s U Test).

Variables	Test Type	Mean Results for Analyzed Groups				*p* Value
		Depressed Group (27%)		Non-Depressed (73%)		
Pretreatment/treatment data						
Smoking tobacco before RT	-	96%		78%		<0.05
Steroids intake during RT	-	85%		58%		<0.05
		mean	median	mean	median	
Intensity of pain of head/neck (0–10)	VAS	4.7	5	2.6	2	<0.001
Intensity of shoulder pain (0–10)	VAS	4.4	5	2	2	<0.001
**Functional scores (0–100)**	QLQ-C30					
Global health status QL2		41.3	45	66.2	66.7	<0.000001
Physical functioning PF		60	60	74.2	80	<0.001
Emotional functioning EF		44.7	41.7	73.7	75	<0.000001
Cognitive functioning CF		65.9	66.7	77.4	83.3	<0.05
Social functioning SF		70.2	66.7	85	100	<0.05
**Symptom scores (0–100)**						
Fatigue FA		53.9	55.6	37.6/	33.3	<0.01
Pain (frequency) PA		53.2	50	20.9	16.7	<0.000001
Dyspnea DY		28	33.3	15.9	0	<0.05
Insomnia SL		58.8	66.7	27.5	33.3	<0.001
Appetite loss AP		44.7	33.3	21	33.3	<0.01
**Symptom scores (0–100)**	H&N35					
HN pain (frequency) HNPA		40.4	33.3	21	16.7	<0.001
Swallowing problems HNSW		40.7	33.3	18.4	8.3	<0.001
Speech HNSP		38.3	33.3	23.4	22.2	<0.05
Social eating HNSC		40.7	26.7	21.7	6.7	<0.01
Social contact HNSO		29.6	33.3	13.7	16.7	<0.001
Less sexuality HNSX		53.6	50	29.9	16.7	<0.05
Opening mouth HNOM		45.7	33.3	16.7	0	<0.01
Dry mouth HNDR		81.5	100	55	33.3	<0.01
Sticky saliva HNSS		74.1	100	52.9	33.3	<0.05
Coughing HNCO		53.1	66.7	36.8	33.3	<0.05
Feeling ill HNFI		57.7	33.3	24.8	33.3	<0.0001
Painkillers HNPK		74.1	100	34.8	0	<0.01
Weight loss HNWL		44.4	0	12.7	0	<0.01

## Data Availability

Data are contained within the article.
